# Evaluation of Biocontrol Agents Against Root-Knot Nematode (*Meloidogyne incognita*) in Cucumber (*Cucumis sativus*) Under Greenhouse Conditions

**DOI:** 10.3390/plants15131979

**Published:** 2026-06-26

**Authors:** Win Lai Lai Swe, Ferenc Tóth, Márta Ladányi, József Fail

**Affiliations:** 1 Department of Entomology, Institute of Plant Protection, University of Agriculture and Life Sciences, Ménesi út 44., H-1118 Budapest, Hungary; winlailaiswe1@gmail.com (W.L.L.S.); fail.jozsef@uni-mate.hu (J.F.); 2 Hungarian Research Institute of Organic Agriculture, Ráby Mátyás u. 26., H-1038 Budapest, Hungary; ferenc.toth@biokutatas.hu; 3 Department of Applied Statistics, Institute of Mathematics and Basic Science, University of Agriculture and Life Sciences, Villányi út 29-43., H-1118 Budapest, Hungary

**Keywords:** *Meloidogyne incognita*, *Cucumis sativus* L., microorganisms, biological control

## Abstract

Root-knot nematode (*Meloidogyne incognita*) is a significant challenge in cucumber (*Cucumis sativus* L.) production under protected cultivation systems. This study aimed to evaluate the effects of four microbial biocontrol agents (*Trichoderma asperellum*, *Beauveria bassiana*, *Fusarium proliferatum,* and *Bacillus mojavensis*) on cucumber growth and root gall formation. Two greenhouse experiments (spring and summer 2023) were conducted using potted plants inoculated with 100 g of nematode-infected soil. All treatments improved seed germination compared with the untreated control, particularly *B. mojavensis* and *T. asperellum*. During vegetative growth, *F. proliferatum* and *B. mojavensis* produced higher biomass in the spring experiment, while *T. asperellum* enhanced root development. Treatment with *T. asperellum* reduced root galling by 45% in the spring and 31% in the summer experiment, although these differences were not statistically significant. In the chemotaxis assay, the different microbial biocontrol agents showed variation in the chemotaxis index (CI), ranging from 0 to 0.12 among treatments. Overall, microbial treatments mainly enhanced plant growth under nematode stress, while effects on root galling were limited.

## 1. Introduction

Growing high-value horticultural crops in polyhouses has increased in recent years due to improved environmental control and higher yield potential [1,2]. However, the intensive cultivation systems characterized by continuous cropping, high fertilizer input, frequent irrigation, elevated temperatures, and high humidity create favorable conditions for soil-borne diseases, especially plant-parasitic nematodes (PPNs) [3]. Globally, PPNs cause annual economic losses estimated between USD 100–175 billion [4,5,6].

Among PPNs, root-knot nematodes (*Meloidogyne* spp.) are widely distributed with more than 105 described species worldwide [7]. *Meloidogyne incognita* is one of the most damaging species in protected cultivation systems in Hungary, particularly in heated polyhouses, where it causes significant yield losses in vegetable crops such as cucumber (*Cucumis sativus* L.) [8]. Root-knot nematodes (RKNs) have four larval stages between the egg and adult stages, with intervening molts. The female lays eggs in the surrounding area, and the life cycle can be completed within 3 to 4 weeks under optimal conditions. However, under unfavorable conditions, nematodes can enter diapause or quiescence for survival [9]. Second-stage juveniles (J2) hatch in the soil and penetrate the roots, where they induce giant cell formation in the vascular cylinder. This leads to abnormal cell proliferation and gall formation, resulting in reduced root function and aboveground symptoms such as wilting, stunting, and chlorosis [9,10,11].

Given the economic losses caused by *M. incognita*, various management strategies have been employed, including chemical control, crop rotation, soil fumigation, solarization, resistant crop plants, and the use of microorganisms [12,13]. Chemical nematicides have traditionally been used to manage root-knot nematode infestations in high-value crops. However, their environmental risks and limited sustainability have increased interest in alternative approaches [14,15]. As an alternative, biocontrol agents not only control root-knot nematodes but also enhance plant growth and may generate systemic defense in plants against biotic stresses [16]. Various biocontrol agents, including fungi, bacteria, and predatory organisms, have been studied for nematode management. Fungi and bacteria act through mechanisms such as antibiosis, parasitism, competition, and induction of plant defense responses [17,18,19].

Research on using beneficial microorganisms to control plant-parasitic nematodes remains limited, particularly regarding the combined effects on plant growth promotion, nematode suppression, and nematode behavior under uniform greenhouse conditions with standardized inoculum from a cucumber production system. Studies that connect greenhouse performance with laboratory-based chemotaxis responses are particularly limited. In this study, microbial biocontrol agents were provided by a commercial biological protection company. The agents included two well-established nematode antagonists, *Trichoderma asperellum* and *Beauveria bassiana*, which are widely recognized for their ability to promote plant growth and exhibit biocontrol activity. Additionally, the study incorporated two less-explored microbial agents: a bacterial strain, *Bacillus mojavensis,* and a non-pathogenic endophytic fungus, *Fusarium proliferatum*. Although some *Fusarium* species are plant pathogens, certain non-pathogenic endophytic isolates can promote plant growth and have biocontrol properties [20].

Therefore, this study evaluated the effects of these four microbial biocontrol agents in cucumber under greenhouse conditions and in laboratory chemotaxis assays. The objectives were to assess their influence on seed germination, plant growth, biomass, and root gall formation under *Meloidogyne incognita* stress, to determine their effects on juvenile chemotactic behavior, as well as to compare responses across two seasonal experiments. It was hypothesized that microbial agents would enhance plant growth and biomass, reduce root gall formation, and influence the chemotactic behavior of second-stage juveniles (J2) under controlled conditions.

## 2. Material and Methods

### 2.1. Experimental Site Description and Source

All experiments were conducted in 2023 under greenhouse conditions at the Research Field of the Hungarian University of Agriculture and Life Sciences (MATE), Budapest-Soroksár, Hungary. Nematode-infested soil samples were collected from a commercial cucumber greenhouse located in Csány, Heves County, Hungary (47°39′22.6″ N, 19°49′29.4″ E). The site had been under continuous cucumber monoculture for more than 25 years and was naturally infested with root-knot nematodes (*Meloidogyne incognita*). Four microbial biocontrol agents were evaluated: *Trichoderma asperellum, Beauveria bassiana, Fusarium proliferatum,* and *Bacillus mojavensis*. All microorganisms were provided by Bioved 2005 Kft. (Pinkamindszent, Hungary). Seeds of the cucumber (*Cucumis sativus* L.) cv. ‘Vert Petit de Paris’ were obtained from the BioKiskert Seed Company (Budapest, Hungary).

### 2.2. Experimental Design and Setup

The study consisted of two independent greenhouse experiments conducted in the spring and summer of 2023 (Table 1).

**Figure 1:**
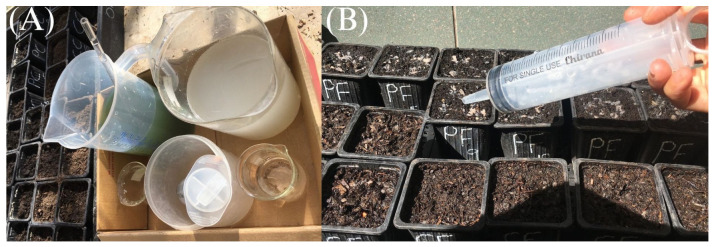
Preparation of material for biocontrol agent application: (**A**) mixture of biocontrol inoculum suspension, (**B**) 30 mL of each specific inoculum applied to nursery pots.

Each experiment included 50 plants arranged in a completely randomized design with five treatments (four microbial treatments and one untreated control), utilizing ten replicate plants per treatment. Microbial biocontrol agents were maintained in Petri dishes at room temperature until suspension preparation. According to supplier information, each Petri dish contained approximately 1 × 10^10^ microbial propagules (fungal conidia or bacterial cells/spores depending on the microorganism). To prepare the inoculum, sterile distilled water was added to each Petri dish, and the agar surface was gently scraped using sterile teaspoons. *Bacillus mojavensis* and *Trichoderma asperellum* were readily suspended in distilled water, whereas *Beauveria bassiana* and *Fusarium proliferatum* required homogenization using a hand mixer to ensure uniform dispersion. The resulting suspensions were combined and adjusted to approximately 480 mL total volume in a sterile container. Each treatment suspension was applied at 30 mL per pot, corresponding to approximately 1.25 × 10^9^ microbial propagules per pot (Figure 1). A total of 16 pots per treatment (10 experimental and 6 reserve pots) were prepared. Control plants received an equal volume of sterile distilled water. Three cucumber seeds were sown into 7 × 7 × 8 cm plastic nursery pots. At the two-leaf stage, the seedlings were thinned to one plant per pot. Seedling trays were maintained under greenhouse conditions until transplanting.

**Figure 2:**
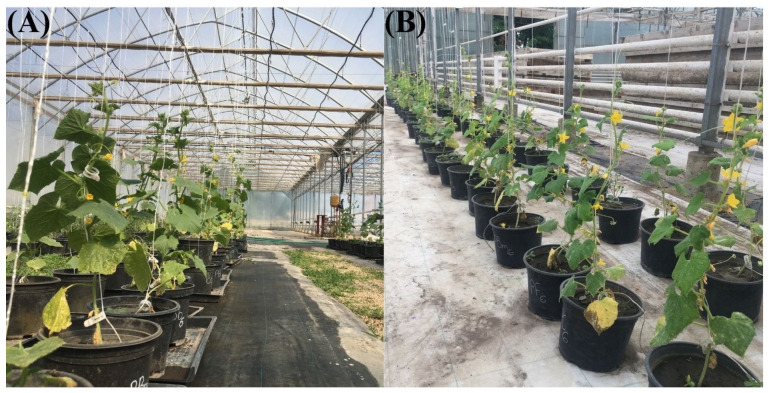
Overview of the experimental set-up in the greenhouse. (**A**) Experiment 1 (2023 spring), (**B**) Experiment 2 (2023 summer).

Approximately 30 days after sowing, uniform seedlings were transplanted into 5-L plastic pots (23 × 18 cm) filled with sterilized sandy loam soil (82% sand, 6% clay, 12% silt). Each pot was amended with 100 g of naturally nematode-infested soil collected from the Csány site. The infested soil was thoroughly mixed into the potting substrate to ensure a uniform distribution of nematodes among experimental units. Although initial nematode density per pot was not quantified, homogenization of the infested soil was performed to minimize variability in nematode pressure; therefore, substantial differences among treatments were not expected. Further randomization was not possible due to the constraints of plant growth and support structures. Data were recorded weekly from germination until harvest (Figure 2).

### 2.3. Assessing the Chemotaxis Index of Biocontrol Agents on Meloidogyne incognita

**Figure 3:**
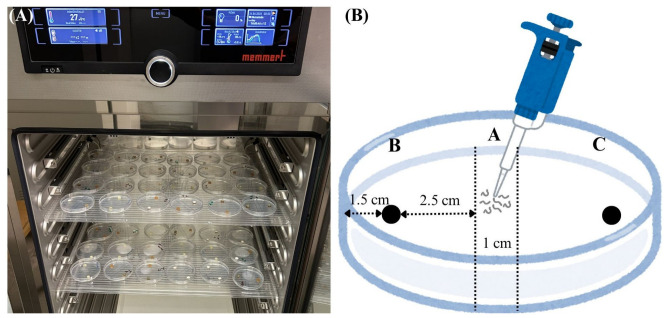
In vitro assay for evaluating chemotactic response against *Meloidogyne incognita*. (**A**) Temperature-adjusted incubation chamber. (**B**) Schematic representation of counting second-stage juveniles (J2) in a Petri dish. B: Active zone; A: neutral zone; C: control zone.

The chemotaxis index of the selected biocontrol agents was evaluated following the protocol of Wang et al. [21], with modifications. The treatments included (I) *T. asperellum*; (II) *B. bassiana*; (III) *F. proliferatum*; (IV) *B. mojavensis*; (V) a positive control; and (VI) a negative control (Figure 3). Each Petri dish (9 cm diameter) was filled with 2% water agar and divided into three zones: middle (1 cm) = neutral zone (A); left (1.5 cm from the border) = active zone (B); right (1.5 cm from the border) = control zone (C). Approximately 20 μL of a suspension containing ~100 J2 of *M. incognita* was applied to the center of the neutral zone (A). A 9-mm agar plug of the biocontrol agents was placed in the active zone (B), while the control zone (C) received only the corresponding agar medium. In the positive controls, the same isolate was placed in both zones, whereas the negative controls contained only the corresponding agar medium. Then, the plates were sealed and kept in a temperature-controlled chamber in the dark for 24 h. Following incubation, the number of J2 in each zone was recorded to calculate the antagonistic activity.

The chemotaxis index (CI) was calculated as

CI = (B − C)/(B + C)

where

C = Number of J2 in the control zone.

B = Number of J2 in the active zone.

Interpretation of CI values:

- CI ≥ 0.2 → Highly attractive.
- 0.1 ≤ CI < 0.2 → Slightly attractive.
−0.1 ≤ CI < 0.1 → Random response.
−0.2 < CI < −0.1 → Slightly repellent.
- CI ≤ −0.2 → Highly repellent.

### 2.4. Data Collection

Plant growth parameters, including plant height (cm), total number of leaves (pc), and number of flowers (pc), were recorded weekly throughout the experimental period. Plant height was measured from the soil surface to the top of the shoots. All plants were harvested 45 days after transplanting, and measurements were taken for fresh plant weight (g/plant), fresh root weight (g/plant), dry root weight (g/plant), and root length (cm). Subsequently, the plants were carefully uprooted, and the roots were thoroughly cleaned to remove soil particles. To determine the root gall index (RGI), which indicates the severity of root-knot nematode (*M. incognita*) infestation, galls were counted in ten different microscopic fields of view per pot under a stereo microscope at a consistent, minimum magnification. Microscopic observations were conducted using the SZM-400 BT Stereo Zoom Microscope at a lower magnification to identify the number of M. incognita root galls, and these observations were conducted at the laboratory of MATE.

### 2.5. Data Analysis

All analyses were conducted in R version 4.5.2 [22], and figures were generated using the ‘ggplot2’ package [23].

The germination rate of cucumber seeds was evaluated under five treatment levels: B. bassiana (Bb), *B. mojavensis* (Bm), *F. proliferatum* (F), T. asperellum (T), and the untreated control (C) across two experiments. To evaluate seed germination success, we constructed a generalized linear model (GLM) using a binomial distribution with a logit link function. The experimental unit was the pot, with the response variable defined as the two-column matrix of successful germinations and failures (out of three seeds planted per pot). Treatment (5 levels: Bb, Bm, F, T, and untreated control C), Experiment (2 levels: spring and summer 2023), and their two-way interaction were treated as fixed effects. Model assumptions were evaluated using simulated quantile residuals via the DHARMa package [24]. The Kolmogorov–Smirnov test confirmed no significant deviation from uniformity (D = 0.071, p = 0.70), and the nonparametric dispersion test showed no evidence of overdispersion or underdispersion (dispersion ratio = 0.81, p = 0.20). Because treatment *B. mojavensis* (Bm) achieved a 100% germination rate in the spring trials (Experiment 1), standard maximum likelihood estimation suffered from complete separation (Hauck–Donner effect [25,26]). To obtain unbiased parameter estimates, stable standard errors, and valid confidence intervals, we implemented Firth’s penalized likelihood logistic regression [27] via the brglm2 package [28]. Post hoc pairwise comparisons among treatments within each experiment were conducted on the log-odds scale using the emmeans package [29], with *p*-values adjusted for multiple comparisons using Tukey’s HSD method.

To account for repeated weekly measurements of plant height, number of leaves, number of flowers and number of branches on the same plants over time, two distinct modeling frameworks were applied to evaluate treatment differences across both the two experiments (spring and summer 2023). First, linear mixed-effects models (LMMs) were used to analyze repeated-measures variables across their respective experimental timelines: plant height in spring (9 weeks) and summer (9 weeks); leaf production (NoLeaves) in spring (9 weeks) and summer (9 weeks); flower production (NoFlowers) in spring (4 weeks) and summer (8 weeks), and branching (NoBranches) in spring (4 weeks). For these LMMs, the models were fitted using the lme4 [30] and lmerTest [31] in R. Treatment (five levels: Bb, Bm, F, T, and untreated control C), time (treated as a categorical factor), and their interaction were specified as fixed effects. To model the repeated-measures covariance structure, prevent pseudo replication, and control for plant-specific variation, a random intercept was assigned to individual plant identity (Plant_ID). Denominator degrees of freedom and *p*-values for fixed effects were approximated using Satterthwaite’s method. Second, one-way ANOVA was used to evaluate the summer experiment for branching (NoBranches), which was measured using a general linear model (lm()) at a single, final time point.

Standardized residuals from both the overall LMMs and the standard linear model were extracted to verify underlying statistical assumptions: (1) Normality was checked by calculating the skewness and kurtosis of the residuals. Residual normality was accepted if the values fell within strict conservative thresholds (below 2 for skewness and below 4 for kurtosis). (2) Homoscedasticity was checked globally across treatment levels and weeks using Levene’s test via the car package [32], with time treated as a categorical factor.

To resolve localized temporal treatment dynamics for the repeated-measures variables, separate weekly one-way ANOVAs were executed for each measurement interval using Bonferroni’s Type I error adjustment. Within each weekly subset (as well as for the single-time-point summer branching data), treatment homoscedasticity was verified using Levene’s test. When homoscedasticity was satisfied (*p* > 0.05), Tukey’s HSD test was performed for pairwise comparisons. When it was violated (*p* < 0.05), the robust Games–Howell post hoc test was applied via the rstatix package [33] to control for Type I error inflation.

A one-way MANOVA model was used to compare the fresh and dry plant and root weights (g), and root length (cm) based on the treatment factor, followed by univariate ANOVA for each variable. To ensure the reliability of the results, Bonferroni’s Type I error correction was employed. The normality of all model residuals was accepted on the basis of the absolute values of skewness and kurtosis being below 1.0. Furthermore, the homogeneous groups were separated employing Tukey’s HSD post hoc test, as the homogeneity of variances was accepted by Levene’s test (*p* > 0.05).

Root gall formation was assessed by counting the number of galls on infected roots. To model the severity of root-knot nematode infestation, Root Gall Index (RGI) values, representing the average gall count across ten different microscopic locations per pot, were analyzed using a generalized linear mixed model (GLMM) with a Gamma distribution and a log-link function. Treatment (5 levels: Bb, Bm, F, T, and untreated control C), Season (2 levels: spring and summer 2023), and their two-way interaction were treated as fixed effects. To account for the repeated-measures design (measuring the same physical pots across each individual experiment), a random intercept for each individual pot (Unique_Pot_ID) was included. Model diagnostics were evaluated using simulated quantile residuals via the DHARMa package [24]. The Kolmogorov–Smirnov test confirmed no significant deviation from uniformity (D = 0.086, *p* = 0.450), and the nonparametric dispersion test confirmed no significant overdispersion or underdispersion (dispersion ratio = 0.84, *p* = 0.432). The GLMM was fitted using the lme4 package [30], and post hoc pairwise comparisons among treatments within each experiment were conducted on the log-odds scale using the emmeans package [29], with *p*-values adjusted for multiple comparisons using Tukey’s HSD method.

To evaluate the antagonistic efficacy while respecting the nested design of the assay (Petri dishes nested within temporal daily blocks), data were analyzed using generalized linear mixed models (GLMMs) with a binomial distribution and a logit link function.

The response variable was modeled as the joint binomial counts of individuals choosing either the active zone or the control zone within each Petri dish. Preference was evaluated individually for each treatment level. The intercept of the model represented the log-odds of selecting the active zone relative to a neutral 50:50 probability. To control for temporal and technical block effects, experimental runs (k = 7) and unique individual Petri dish identifiers (Dish_ID) were incorporated as random intercept effects. Treating the individual Petri dish (Dish_ID) as a random grouping factor structurally corrected the models for potential overdispersion.

Model diagnostics, including tests for overdispersion and residual distribution patterns, were formally evaluated using simulated quantile residuals via the DHARMa package [24] in R. Standardized effect sizes are reported as odds ratios (ORs) alongside 95% confidence intervals (CIs).

## 3. Results

### 3.1. Influence of Biocontrol Agents on Germination Rate (%)

**Figure 4:**
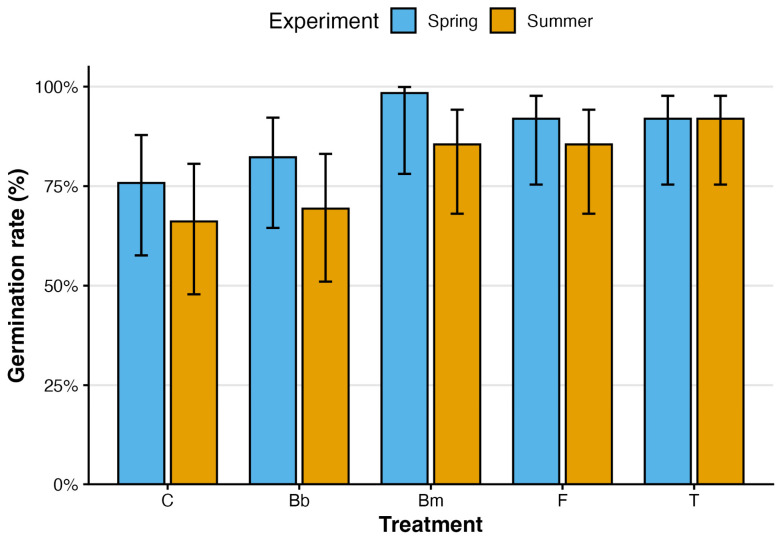
Estimated seed germination probabilities (%) across five treatment levels (biocontrol agents *Beauveria bassiana* (Bb), *Bacillus mojavensis* (Bm), *Fusarium proliferatum* (F), *Trichoderma asperellum* (T), and untreated control (C) during the spring and summer experiments in 2023. Bars represent the estimated marginal means back-transformed from the logit scale, and error bars denote the asymptotic 95% confidence intervals. Estimation was performed using Firth’s penalized likelihood logistic regression (N = 10 pots per treatment–experiment combination, with 3 seeds per pot) to resolve complete separation caused by 100% germination in the spring Bm group. No statistically significant pairwise differences were detected within either experiment (Tukey’s HSD, *p* > 0.05).

Mean germination rates (probabilities) and their asymptotic 95% confidence intervals (CIs) estimated via Firth’s penalized likelihood are summarized in Figure 4. In the spring season (Experiment 1), estimated germination rates were high across all groups, ranging from 75.8% (95% CI: 57.6–87.8%) in the untreated control (C) group to 98.4% (95% CI: 78.1–99.9%) in the Bm group. In the summer trials (Experiment 2), germination rates were slightly lower, ranging from 66.1% (95% CI: 47.8–80.6%) in the untreated control (C) group to 91.9% (95% CI: 75.4–97.7%) in the T group. Despite these numerical differences, pairwise comparisons of treatments within each experiment yielded no statistically significant differences under Tukey’s adjustment (all *p* > 0.05). In spring, the highest difference was observed between the Bm and untreated control samples (odds ratio [OR] = 19.47, *p* = 0.28), while other contrasts showed negligible statistical effects (e.g., untreated control vs. Bb: OR = 1.48, *p* = 0.97; F vs. Bb: OR = 0.41, *p* = 0.81). Similarly, in summer, treatment differences remained statistically non-significant (e.g., Bm vs. untreated control: OR = 3.02, *p* = 0.43; T vs. untreated control: OR = 0.17, *p* = 0.15).

### 3.2. Effect of Biocontrol Agents on Cucumber (Cucumis sativus L.) Plant Growth

The study evaluated the effectiveness of biocontrol agents and their nematicidal effects on cucumber plants by examining morphological parameters, including plant height (cm), number of leaves, number of flowers, and number of branches (Table 2, Supplementary Material Table S1). This assessment performed across two separate greenhouse experiments in 2023: Experiment 1 (2023 spring) and Experiment 2 (2023 summer).

Plant height

The overall linear mixed-effects model (LMM) for the spring (Experiment 1) revealed a highly significant main effect of time on plant height (F(8, 360) = 523.91, *p* < 0.001), whereas the main effect of treatment was marginally non-significant (F(4, 45) = 2.37, *p* = 0.07). However, a highly significant treatment-by-time interaction was observed (F(32, 360) = 2.25, *p* < 0.001), indicating that the height differences between treatment groups emerged progressively over time.

The plant-to-plant random variation accounted for a variance of 70.32 (SD = 8.39), with a residual variance of 140.72 (SD = 11.86). For the summer (Experiment 2), the LMM showed highly significant overall effects for all factors, including treatment (F(4, 45) = 4.83, *p* < 0.01), time (F(8, 360) = 363.35, *p* < 0.001), and the treatment-by-time interaction (F(32, 360) = 2.72, *p* < 0.001). In the summer experiment, the plant-to-plant random variance was lower at 16.75 (SD = 4.09), with a residual variance of 56.28 (SD = 7.50).

In the spring experiment, weekly one-way ANOVAs confirmed that treatment effects remained non-significant during the early growth phase of weeks 1–3 (*p* > 0.05). A marginal treatment trend began to manifest at week 4 (F(4, 45) = 2.26, *p* = 0.08), but statistical significance was not established until the final measurement stages. By week 9, treatment differences became highly significant (F(4, 45) = 4.22, *p* < 0.01). Pairwise comparisons at week 9 separated the treatment means, with letter groupings progressing from the lowest mean plant height to the highest (Table 2, Supplementary Material Table S1).

In the summer experiment, treatment effects were non-significant during weeks 1–2 (*p* > 0.05) but emerged rapidly by week 3 (F(4, 45) = 5.28, *p* < 0.01) and remained significant through the final week of the trial (week 9: F(4, 45) = 3.30, *p* < 0.05). Post hoc testing partitioned the treatments into distinct homogeneous subsets starting from week 3, highlighting the diverging growth rates among the five factor levels as the experiment progressed (Table 2, Supplementary Material Table S1).

Number of leaves

The overall LMM for the spring (Experiment 1) indicated a highly significant main effect of time on leaf production (F(8, 360) = 597.00, *p* < 0.001), while the main effect of treatment was marginally non-significant (F(4, 45) = 2.37, *p* = 0.07). However, the treatment-by-time interaction was highly significant (F(32, 360) = 1.96, *p* < 0.01). The plant-to-plant random variation accounted for a variance of 1.65 (SD = 1.28), with a residual variance of 3.84 (SD = 1.96). For the summer (Experiment 2), all factors in the LMM were highly significant, including treatment (F(4, 45) = 6.85, *p* < 0.001), time (F(8, 360) = 931.54, *p* < 0.001), and their interaction (F(32, 360) = 3.17, *p* < 0.001). Under summer conditions, the plant-to-plant random variance was lower at 0.56 (SD = 0.75), with a residual variance of 1.34 (SD = 1.16).

In the spring experiment, weekly one-way ANOVAs showed no significant differences among treatments during the early stages of weeks 1–8 (*p* > 0.05). However, treatment effects became highly significant by week 9 (F(4, 45) = 3.94, *p* < 0.01), driven by a pronounced decrease in leaf numbers in untreated control plants (Table 2 and Table S1).

In the summer experiment, weekly one-way ANOVAs showed no significant treatment differences during the initial weeks (week 1: *p* = 0.76; week 2: *p* = 0.48). A brief significant difference was recorded at weeks 3–4 (F(4, 45) > 4.07, *p* < 0.01), followed by non-significant values in week 5 (*p* > 0.05). Consistently significant treatment effects were established from weeks 6 through week 9 (week 6: F(4, 45) = 2.94, *p* < 0.05; week 7: F(4, 45) = 7.02, *p* < 0.001; week 8: F(4, 45) = 5.03, *p* < 0.01; week 9: F(4, 45) = 5.84, *p* < 0.001). Post hoc analyses during this final phase indicated that leaf production was consistently and severely suppressed in untreated control plants compared to the other groups, showing significantly fewer leaves than the reference intercept throughout weeks 7–9 (Table 2, Supplementary Material Table S1).

Number of flowers

The overall LMM for the spring (Experiment 1) revealed a highly significant main effect of time on flower production (F(3, 135) = 161.10, *p* < 0.001). However, the overall main effect of treatment was non-significant (F(4, 45) = 1.66, *p* = 0.18), and no significant interaction was detected between treatment and time (F(12, 135) = 1.50, *p* = 0.13). Plant-to-plant random variation accounted for a variance of 10.57 (SD = 3.25), with a residual variance of 7.47 (SD = 2.73). For the summer (Experiment 2), the LMM showed a highly significant main effect of treatment (F(4, 45) = 4.47, *p* < 0.01) and time (F(6, 275) = 27.66, *p* < 0.001). The interaction between treatment and time was non-significant (F(24, 275) = 1.13, *p* = 0.30). Plant-to-plant random variance was 1.50 (SD = 1.23), with a residual variance of 16.62 (SD = 4.08).

In the spring experiment, weekly one-way ANOVAs confirmed that treatment effects remained statistically non-significant throughout the entire 4-week trial (weeks 6–9) when flowers were observable (week 6: F(4, 45) = 1.08, *p* = 0.38; week 7: F(4, 45) = 2.07, *p* = 0.10; week 8: F(4, 45) = 1.53, *p* = 0.21; week 9: F(4, 45) = 1.53, *p* = 0.21). Consequently, while flower production increased over time across all groups, no treatment-induced differences were established during the spring experiment (Table 2, Supplementary Material Table S1).

In the summer experiment, weekly one-way ANOVAs identified significant differences at multiple time points. During the earliest phases when flowers were observed (weeks 3–4), a significant treatment effect was detected (F(4, 45) = 5.18, *p* < 0.01). During the mid-phases (weeks 5–7), treatment effects became temporarily non-significant (*p* > 0.05). During the late-phases (weeks 8–9), treatment effects re-emerged as marginally significant in week 8 (F(4, 45) = 2.56, *p* = 0.05) and became fully significant by week 9 (F(4, 45) = 2.81, *p* < 0.05). Where significant differences were established, pairwise comparisons indicated that flower production was consistently suppressed in untreated control samples compared to the other groups (Table 2, Supplementary Material Table S1).

Number of branches

For the spring (Experiment 1), the overall LMM revealed a highly significant main effect of time on the number of branches (F(3, 135) = 18.29, *p* < 0.001). The main effect of treatment was non-significant (F(4, 45) = 2.22, *p* = 0.08). No significant interaction was detected between treatment and time (F(12, 135) = 0.73, *p* = 0.7), indicating that branching trends remained relatively parallel among treatment groups over the 4-week period. Plant-to-plant random variation accounted for a variance of 0.61(SD = 0.78), with a residual variance of 1.94 (SD = 1.39). For the summer (Experiment 2, measured at a single final time point), a standard one-way ANOVA was performed. Plant-to-plant variation was modeled within the residual variance of 0.46 (SD = 0.68).

In the spring experiment, weekly one-way ANOVAs confirmed that treatment effects remained statistically non-significant throughout the entire 4-week (weeks 6–9) trial (week 6: F(4, 45) = 0.64, *p* = 0.64; week 7: F(4, 45) = 1.01, *p* = 0.41; week 8: F(4, 45) = 1.81, *p* = 0.14; week 9: F(4, 45) = 1.81, *p* = 0.14). While the average number of branches grew over time across all groups, no distinct treatment-induced differences were statistically established during the spring experiment (Table 2, Supplementary Material Table S1).

In the summer experiment, the one-way ANOVA at week 9 showed no statistically significant effect of treatment on the number of branches (F(4, 45) = 0.81, *p* = 0.53). The final mean branch counts among the treatment levels ranged from a low of 0.10 ± 0.32 in untreated control plants to a high of 0.60 ± 1.07 in the *Fusarium proliferatum* (F) treatment (Table 2, Supplementary Material Table S1).

### 3.3. Effect of Biocontrol Agents on Plant Biomass

The effectiveness of biocontrol agents on cucumber plant biomass (fresh and dry plant and root weights (g) and root length (cm)) was assessed in two separate greenhouse experiments conducted at different time points (Table 3). The one-way MANOVA models were highly significant for both experiments (Experiment 1: Wilks’ lambda = 0.20, F(24, 140.75) = 3.45, *p* < 0.001; Experiment 2: Wilks’ lambda = 0.36, F(24, 140.75) = 2.02, *p* < 0.01). In Experiment 1 (spring), both fresh plant weight (*F*(4, 45) = 9.59, *p* < 0.001) and dry plant weight (*F*(4, 45) = 7.87, *p* < 0.001) were significantly influenced by the treatments. Post hoc comparisons showed that *F. proliferatum* (F) and *B. mojavensis* (Bm) recorded the highest fresh and dry plant weights, significantly higher than the untreated control (C), while *T. asperellum* (T) displayed intermediate values that were still significantly higher than those of untreated control. Underground biomass traits (fresh root weight, dry root weight, and root length) did not differ significantly among treatments (*F*(4, 45) < 2.31, *p* > 0.05).

In Experiment 2 (summer), treatment effects on aboveground biomass were weaker. Neither fresh plant weight (*F*(4, 45) = 2.24, *p* = 0.08) nor dry plant weight (*F*(4, 45) = 1.20, *p* = 0.32) differed significantly among treatments. Similarly, no significant differences were observed for fresh and dry root weights (*F*(4, 45) < 0.45, *p* > 0.75). However, root length exhibited significant variation across treatments (*F*(4, 45) = 6.41, *p* < 0.001). All agents significantly increased root length compared to the untreated control (from 26.70 cm in untreated control to 45.6 cm in the *T. asperellum* (T) treatment).

### 3.4. Effect of Biocontrol Agents on Meloidogyne incognita Root Gall Index

The effect of biocontrol agents on root gall index (RGI) formation in *Cucumis sativus* was evaluated 45 days after transplanting under greenhouse conditions.

**Figure 5:**
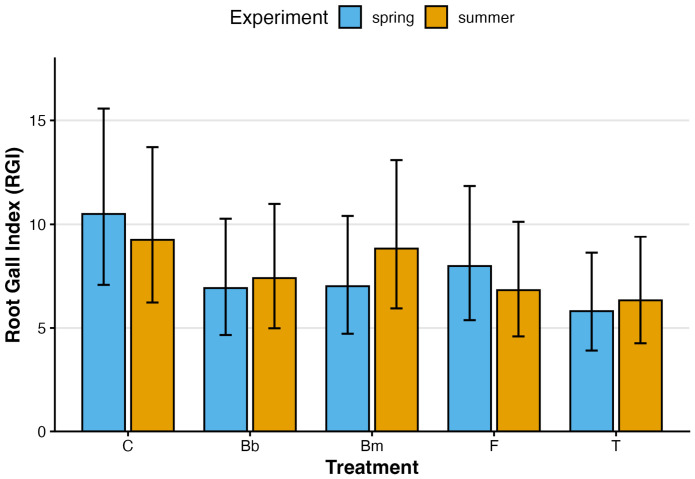
Root Gall Index (RGI) across five treatment levels (*Beauveria bassiana* (Bb), *Bacillus mojavensis* (Bm), *Fusarium proliferatum* (F), *Trichoderma asperellum* (T) and unteated control C) during the spring and summer experiments. Bars represent back-transformed estimated marginal means modeled via a generalized linear mixed model (GLMM) with a Gamma distribution and log-link function. Error bars represent asymptotic 95% confidence intervals. The random intercept for individual pots accounted for the repeated-measures design across both experiments. No statistically significant pairwise differences were found between treatments within either experiment (Tukey’s HSD, *p* > 0.05).

The Gamma GLMM successfully accommodated the right-skewed distribution and variability of the RGI dataset. Back-transformed estimated mean RGI values and their asymptotic 95% confidence intervals (CIs) are illustrated in Figure 5.

In the spring season (Experiment 1), the untreated control (C) group displayed the highest estimated mean RGI at 10.48 (95% CI: 6.93–15.83), followed by *F. proliferatum* (F: mean = 7.93, 95% CI: 5.20–12.09), *B. mojavensis* (Bm: mean = 7.00, 95% CI: 4.64–10.58), and *B. bassiana* (Bb: mean = 6.89, 95% CI: 4.53–10.46). The lowest infestation was observed in the *T. asperellum* (T) treatment group at 5.74 (95% CI: 3.68–8.97). In the summer trials (Experiment 2), RGI values remained generally consistent, ranging from 6.27 (95% CI: 4.05–9.70) in the T group to 9.14 (95% CI: 5.91–14.16) in the untreated control (C) group. Pairwise comparisons between treatments within each experiment revealed that the differences did not reach statistical significance after Tukey’s adjustment (all *p* > 0.05). In spring, the comparison between the untreated control (C) and T treatments represented the largest effect, though it remained statistically non-significant (Ratio = 1.83, *p* = 0.28). All other treatment contrasts within the spring trials were highly non-significant (e.g., Bb vs. Bm: Ratio = 0.98, *p* > 0.999; Bm vs. F: Ratio = 0.88, *p* = 0.99). Similarly, in summer, pairwise treatment differences were non-significant (e.g., C vs. T: Ratio = 1.46, *p* = 0.71; F vs. T: Ratio = 1.08, *p* > 0.999).

### 3.5. Assessing the Chemotactic Responses of Biocontrol Agents on Meloidogyne incognita

**Figure 6:**
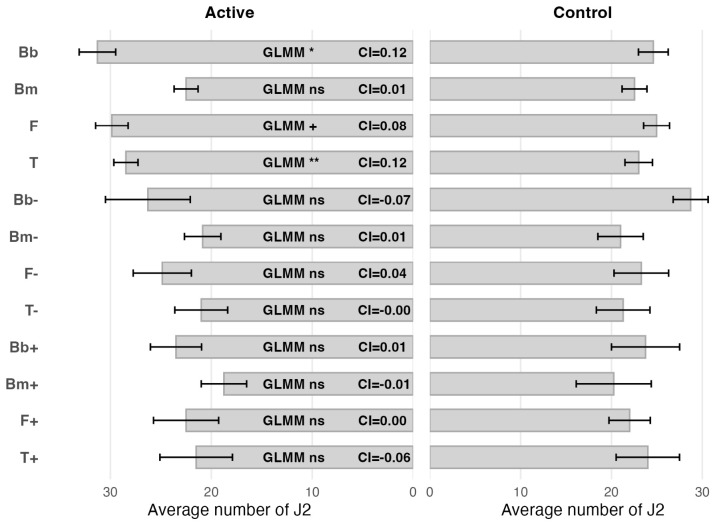
Spatial preference distribution of J2 nematodes across treatments (*Beauveria bassiana* (Bb), *Bacillus mojavensis* (Bm), *Fusarium proliferatum* (F) and *Trichoderma asperellum* (T). Positive and negative symbols (Bb+, Bm+, F+, T+, Bb-, Bm-, F-, T- denote positive control (when the same isolate was placed in both zones) and negative control (when both sides contained only specific agar medium), respectively. Left and right horizontal panels represent the average number of nematodes recovered from the active and control zones of the Petri dishes, respectively. Error bars denote the standard error of the mean (SEM) of replicates. Text labels overlaying the active bars summarize the individual binomial GLMM significance levels (intercept difference from 50:50 probability) along with the calculated mean Chemotaxis Index (CI = (Active – Control)/(Active + Control) when being significant, indicate repellence (CI < 0) or attraction (CI > 0). Significance at + *p* < 0.10; ** *p* < 0.01; * *p* < 0.05; ns = non-significant.

The chemotaxis responses of *M. incognita* J2 nematodes to four biocontrol agents were evaluated after 24 h of exposure using the chemotaxis index (CI). The statistical summaries for all treatment and control groups are detailed in Table 4. Significant differences in nematode distribution between active and control zones were observed for *T. asperellum* and *B. bassiana* (T: Z = 2.74, *p* < 0.01; Bb: Z = 1.97, *p* < 0.05, respectively), choosing the active zone with mean chemotaxis indices CI_T_ = 0.117 and CI_Bb_ = 0.123. The odds of choosing the active zone under T treatment were 1.25 times higher than choosing the control zone (95% CI = [1.066, 1.476]), and 1.27 times higher for Bb (95% CI = [1.001, 1.603]). *F. proliferatum* (F) showed a marginal trend toward active zone preference, (Z = 1.79, *p* = 0.07, OR = 1.20). No significant preference was observed in the *B. mojavensis* (Bm) treatment (Z = 0.03, *p* = 0.98, OR = 1.00). Importantly, none of the negative or positive controls demonstrated a statistically significant spatial preference (all *p* > 0.49, Table 4, Figure 6). The absence of preference in the negative controls confirms that the carrier solvent did not exert any confounding attractive or repellent bias.

## 4. Discussion

Root-knot nematodes (*Meloidogyne incognita*) are among the most important plant-parasitic nematodes affecting cucumber production worldwide. In Hungary, *M. incognita* was first reported by Andrássy [34] and has since become one of the dominant nematode species associated with protected cultivation systems, particularly in greenhouse and polyhouse systems [14]. Growing restrictions on chemical nematicides have increased interest in biological alternatives that may support plant growth and contribute to sustainable nematode management. Therefore, the present study evaluated the effects of *T. asperellum*, *B. bassiana*, *F. proliferatum,* and *B. mojavensis* on cucumber growth and their interactions with *M. incognita* under greenhouse conditions. Overall, several biocontrol treatments improved seed germination and plant growth parameters under nematode-infested conditions. Although numerical reductions in root galling were observed in some treatments, these differences were not statistically significant. Therefore, this study supports the plant growth-promoting potential of selected biocontrol agents rather than conclusive suppression of *M. incognita*.

These biocontrol agents had a positive impact on cucumber seed germination and the initial stages of seedling development. *B. mojavensis* (Bm) enhanced germination in the spring experiment, while *T. asperellum* (T) had a similar effect in the summer experiment. These differences were small but consistent across observations. Early germination and vigorous seedling establishment are important indicators of seed vigor and can contribute to improved crop performance at later growth stages. These beneficial effects may result from enhanced nutrient mobilization, improved rhizosphere microbial activity, and the production of plant growth-promoting metabolites, including phytohormones and other bioactive compounds [19,35]. Similar results have been reported in previous studies: *Trichoderma* spp. and *Bacillus* spp. not only enhanced cucumber seed germination but also led to the development of healthier and more vigorous seedlings [36,37,38].

The growth responses of cucumber plants to biocontrol agents varied among treatments and between experiments, although the differences were generally small. In Experiment 1, *B. mojavensis* (Bm) showed slightly higher values in plant height, leaf number, and flower production compared to other treatments. In Experiment 2, *F. proliferatum* (F) and *T. asperellum* (T) showed small increases in plant height and leaf development. This finding is consistent with previous studies demonstrating that plant growth-promoting rhizobacteria and endophytic fungi may enhance plant development by improving nutrient uptake, hormone production, and modulation of plant defense responses [36,39,40]. *T. asperellum* (T) also improved root growth in Experiment 2, supporting earlier reports that *Trichoderma* spp. stimulate shoot and root development through multiple mechanisms, including hormonal signaling, nutrient mobilization, and siderophore-mediated iron uptake [41,42]. In contrast, *B. bassiana* (Bb) produced limited effects on cucumber growth in both experiments. This finding contradicts previous studies in other crops where *B. bassiana* colonization led to early flowering and increased fruit yield [43]. This suggests that plant responses to biocontrol agents depend on the host and environmental compatibility. The differences between Experiment 1 and Experiment 2 were generally minor, showing only slight variations in the measured plant traits. These variations likely represent typical biological variability observed under greenhouse conditions, rather than significant seasonal or environmental effects.

There was no significant influence on plant biomass among the treatments. However, in Experiment 1, *F. proliferatum* (F) and *B. mojavensis* (Bm) produced slightly higher fresh and dry plant biomass than the untreated control (C), while *T. asperellum* showed intermediate values. In Experiment 2, treatment effects on aboveground biomass were less pronounced; however, all biocontrol agents significantly increased root length relative to the control. Improved root development may enhance water and nutrient acquisition and contribute to overall plant performance. Similar effects have been reported for *Trichoderma* spp., *Bacillus* spp., and certain endophytic fungi, which are known to influence root architecture and nutrient uptake through multiple physiological mechanisms [44,45,46,47]. Although some differences were observed between the spring and summer experiments, the overall responses were broadly similar. The same biocontrol agents frequently ranked among the better-performing treatments across multiple plant growth parameters, while differences were relatively small. Such variation is expected under greenhouse conditions and may reflect normal biological variability, differences in plant development, or minor environmental fluctuations between experimental periods.

Regarding root galling, numerical differences in root gall index (RGI) were observed among treatments in both experiments. The untreated control exhibited the highest galling levels, whereas *T. asperellum* showed the lowest RGI values. When compared to the control, *T. asperellum* was associated with approximately 45% and 31% lower galling levels in the spring and summer experiments, respectively. Although numerical differences were observed among treatments, Tukey’s HSD test revealed no statistically significant pairwise differences in either experiment. Previous studies have reported that *Trichoderma* spp. may suppress *M. incognita* through multiple mechanisms, including competition in the rhizosphere, production of secondary metabolites, parasitism of eggs, and induction of plant defense responses [48,49,50]. These results indicate that *T. asperellum* may have a positive effect against *M. incognita* in different cropping systems, although efficacy may vary depending on host and environmental conditions.

The chemotaxis assay demonstrated that the tested biocontrol isolates influenced the movement of *M. incognita* juveniles (J2). Specifically, *B. bassiana*, *F. proliferatum*, and *T. asperellum* exhibited slight attraction effects, while *B. mojavensis* and the control treatments (both positive and negative) showed no significant impact. Overall, the chemotaxis index (CI) values were generally low; *B. bassiana* (0.12) and *T. asperellum* (0.11) indicate weak attraction, while *F. proliferatum* showed a borderline response (0.09). This suggests that microbial isolates can affect nematode orientation through chemical signaling, likely involving volatile compounds and secondary metabolites produced by biocontrol fungi [51,52]. However, greenhouse experiments conducted in the spring and summer did not show any differences in root galling among treatments. This indicates that under more complex soil and plant conditions, chemotaxis alone is insufficient to change infection outcomes. Therefore, the chemotaxis results should be interpreted as evidence of nematode–microbe interactions rather than as indicators of successful nematode suppression.

The effectiveness of biocontrol agents against plant-parasitic nematodes depends not only on microbial activity but also on the vigor of the host plant responses and the environmental conditions. Although several biocontrol agents improved plant growth traits, their effects on root galling were limited and inconsistent. Similar observations have been reported in cucurbit production systems, where microbial inoculants that suppress *M. incognita* in one crop or environment may perform differently in another [53,54]. Variations between the spring and summer experiments may have been influenced by seasonal differences affecting plant growth, microbial establishment, and plant–microbe interactions. Furthermore, nematode infection can impair photosynthetic activity, nutrient uptake, and overall plant performance, potentially reducing the capacity of plants to benefit from microbial inoculation [55]. Moreover, plants under severe nematode stress may lack the physiological resilience to respond effectively to microbial inoculants, especially if environmental conditions are not optimal for the proliferation and activity of the applied microbes [56]. If microbial agents are not well adapted to the local soil environment or fail to colonize the rhizosphere effectively, their biocontrol potential becomes limited [54,57,58].

Overall, the present study demonstrated that selected biocontrol agents positively influenced cucumber growth and overall performance under nematode-infested conditions. However, their impact on root galling caused by *M. incognita* was not statistically significant. These findings suggest that the tested biocontrol agents may consistently promote plant growth rather than directly suppressing nematodes. Future studies should include additional measures of nematode reproduction, such as egg mass production, egg counts, and juvenile population densities, together with assessments of microbial colonization and performance under diverse environmental conditions.

## 5. Conclusions

This study evaluated selected microbial biocontrol agents under greenhouse conditions and in laboratory chemotaxis assays to assess their effects on cucumber (*Cucumis sativus* L.) growth and interactions with *Meloidogyne incognita*. Several treatments positively influenced seed germination and early seedling development, with *B. mojavensis* resulting in higher germination in the spring experiment and *T. asperellum* in the summer experiment. Treatment effects on vegetative growth were generally modest; however, *B. mojavensis*, *T. asperellum*, and *F. proliferatum* were associated with relatively higher values for plant height, leaf number, biomass, and root development compared with the untreated control.

Numerical reductions in Root Gall Index (RGI) were observed in some treatments, particularly with *T. asperellum*; however, these differences were not statistically significant in either experiment. The chemotaxis assay demonstrated that *B. bassiana*, *F. proliferatum*, and *T. asperellum* influenced the movement of second-stage juveniles of *M. incognita*, whereas *B. mojavensis* showed no clear response. Nevertheless, the chemotaxis responses observed under laboratory conditions were not associated with reduced root galling under greenhouse conditions. Although minor differences were observed between the spring and summer experiments, the overall treatment responses were comparable across both experimental periods.

Overall, the results indicate that the tested microbial agents primarily enhanced cucumber growth in nematode-infested conditions, although their effect on root galling caused by *M. incognita* was limited. Future studies should assess additional nematode parameters such as egg production, counts, and juvenile densities, alongside microbial colonization in the rhizosphere. Validation under varying nematode densities and conditions, along with longer-term greenhouse or field studies, would enhance the evaluation of their biocontrol potential.

## Figures and Tables

**Table 1:** Experimental timeline and total crop duration of each experiment.

Experiment	Seed Sowing Date	Transplant Date	Harvest Date	Seedling Stage (Days)	Post-Transplant Stage (Days)	Total Duration (Days)
Experiment 1	6 May 2023	5 June 2023	20 July 2023	30	45	75
Experiment 2	20 July 2023	18 August 2023	2 October 2023	29	45	74

**Table 2:** Means and standard deviations of morphological parameters (plant height (cm), number of leaves (pc), number of flowers (pc) and number of branches (pc)) of cucumber plants (n = 10) under *Meloidogyne incognita* infection depending on biocontrol agents *Beauveria bassiana* (Bb), *Bacillus mojavensis* (Bm), *Fusarium proliferatum* (F), *Trichoderma asperellum* (T), and untreated control treatment during the spring and summer experiments in 2023. Different letters show significant differences resulting from pairwise comparisons that were performed using Tukey’s HSD test or the Games–Howell test, conditioned on the homoscedasticity of each weekly subset as determined by Levene’s test (*p* < 0.05). Data of weeks 4 and 9 are shown. Data of all weeks are provided in Supplementary Material Table S1.

Measurements	Treatment	Weeks 4 and 9 of Experiment 1		Weeks 4 and 9 of Experiment 2	
Time of Measurements		7 June 2023	20 July 2023	25 August 2023	29 September 2023
Plant Height (cm)	Control	20.83 ± 2.60 ^a^	93.60 ± 24.77 ^a^	11.41 ± 1.89 ^a^	48.20 ± 17.73 ^a^
	Bb	25.10 ± 8.21 ^ab^	109.60 ± 18.70 ^ab^	13.34 ± 2.02 ^ab^	69.00 ± 20.47 ^ab^
	Bm	27.09 ± 4.44 ^b^	134.00 ± 23.10 ^b^	15.22 ± 2.71 ^bc^	70.70 ± 21.85 ^ab^
	F	24.33 ± 3.80 ^ab^	128.80 ± 27.21 ^b^	17.01 ± 3.98 ^c^	77.80 ± 21.03 ^b^
	T	23.53 ± 2.82 ^ab^	120.42 ± 29.16 ^ab^	13.51 ± 1.40 ^ab^	72.50 ± 17.42 ^ab^
Number of Leaves	Control	3.8 ± 0.63 ^a^	17.3 ± 3. 56 ^a^	4.7 ± 0.67 ^a^	13.1 ± 3.38 ^a^
	Bb	4.7 ± 0.95 ^a^	22.0 ± 3.59 ^ab^	5.1 ± 0.74 ^ab^	15.9 ± 1.37 ^ab^
	Bm	4.6 ± 0.52 ^a^	23.5 ± 3.21 ^b^	5.1 ± 0.57 ^ab^	16.5 ± 2.07 ^b^
	F	4.5 ± 0.71 ^a^	23.4 ± 4.09 ^b^	5.6 ± 0.70 ^b^	17.6 ± 2.12 ^b^
	T	4.6 ± 0.70 ^a^	22.0 ± 5.37 ^ab^	5.1 ± 0.57 ^ab^	16.6 ± 1.65 ^b^
Number of Flowers	Control		9.00 ± 3.33 ^a^	1.2 ± 1.03 ^a^	5.4 ± 3.17 ^a^
	Bb		11.3 ± 4.64 ^a^	2.2 ± 1.32 ^ab^	10.4± 6.13 ^a^
	Bm		14.7 ± 4.99 ^a^	3.4 ± 1.26 ^b^	9.9 ± 3.41 ^a^
	F		12.6 ± 5. 56 ^a^	3.4 ± 1.84 ^b^	9.7 ± 2.95 ^a^
	T		11.2 ± 7.39 ^a^	1.9 ± 1.10 ^ab^	10.3 ± 3.33 ^a^
Number of branches (Shoot)	Control		0.6 ± 1.26 ^a^		0.1 ± 0.32 ^a^
	Bb		1.2 ± 1.48 ^a^		0.6 ± 1.26 ^a^
	Bm		2.1 ± 1.97 ^a^		0.6 ± 0.70 ^a^
	F		2.3 ± 1.64 ^a^		0.5 ± 0.53 ^a^
	T		1.5 ± 1.65 ^a^		0.4± 0.70 ^a^

**Table 3:** Means and standard deviations of biomass production (fresh and dry plant and root weights (g) and root length (cm)) of cucumber under *Meloidogyne incognita* infection depending on biocontrol agents *Beauveria bassiana* (Bb), *Bacillus mojavensis* (Bm), *Fusarium proliferatum* (F) and *Trichoderma asperellum* (T) treatments compared to untreated control (C). Different letters show significant differences (Tukey’s HSD, *p* < 0.05).

Morphology Measurements	Treatment	Experiment 1	Experiment 2
Time of Measurements		20 July 2023	2 October 2023
Fresh Plant Weight (g/plant)	C	54.9 ± 17.01 ^a^	23.06 ± 9.92 ^a^
	Bb	66.7 ± 9.08 ^ab^	30.37 ± 6.91 ^a^
	Bm	86.4 ± 15.56 ^c^	29.59 ± 7.3 ^a^
	F	87.7 ± 11.67 ^c^	31.49 ± 6.87 ^a^
	T	79.2 ± 16.4 ^bc^	31.59 ± 5.8 ^a^
Dry Plant Weight (g/plant)	C	9.18 ± 2.98 ^a^	3.9 ± 1.64 ^a^
	Bb	11.59 ± 2.2 ^ab^	4.6 ± 0.95 ^a^
	Bm	14.72 ± 2.4 ^b^	4.56 ± 1.28 ^a^
	F	14.55 ± 2.20 ^b^	4.82 ± 1.01 ^a^
	T	13.8 ± 3.33 ^b^	4.95 ± 0.80 ^a^
Fresh Root Weight (g/plant)	C	10.83 ± 4.4 ^a^	7.33 ± 4.36 ^a^
	Bb	8.44 ± 3.41 ^a^	6.91 ± 1.95 ^a^
	Bm	10.39 ± 2.55 ^a^	7.91 ± 2.79 ^a^
	F	10.88 ± 3.61 ^a^	7.37 ± 2.39 ^a^
	T	8.89 ± 4.38 ^a^	8.17 ± 2.17 ^a^
Dry Root Weight (g/plant)	C	1.54 ± 0.59 ^a^	0.79 ± 0.44 ^a^
	Bb	1.09 ± 0.39 ^a^	1.01 ± 0.46 ^a^
	Bm	1.50 ± 0.38 ^a^	0.91 ± 0.26 ^a^
	F	1.45 ± 0.53 ^a^	0.91 ± 0.36 ^a^
	T	1.34 ± 0.52 ^a^	0.88 ± 0.28 ^a^
Root Length (cm)	C	33.1 ± 9.31 ^a^	26.7 ± 9.92 ^a^
	Bb	37.85 ± 9.07 ^a^	39.8 ± 9.73 ^b^
	Bm	44.13 ± 8.09 ^a^	38.4 ± 9.52 ^b^
	F	38.62 ± 6.72 ^a^	40.7 ± 3.92 ^b^
	T	33.31 ± 12.89 ^a^	45.6 ± 9.11 ^b^

**Table 4:** Statistical parameters of attraction/repellence analysis. Summarized outputs from the individual binomial generalized linear mixed models (GLMMs) fitted for treatment levels (*Beauveria bassiana* (Bb), *Bacillus mojavensis* (Bm), *Fusarium proliferatum* (F) and *Trichoderma asperellum* (T) (as well as positive and negative controls (Bb+, Bm+, F+, T+, Bb-, Bm-, F-, T-), i.e., when the same isolate was placed in both zones and when both sides contained only specific agar medium, respectively. Mean Chemotaxis Index (CI) values are presented alongside the estimated fixed intercept parameters, which represent log-odds, standard errors (SE), Wald Z-values, and asymptotic *p*-values. Effect sizes are expressed as odds ratios (OR) with Wald-type 95% confidence intervals (CI). An OR value above 1.0 indicates a directional preference for the active zone, whereas an OR value below 1.0 indicates a directional preference for the control zone. Significance levels are denoted as follows: + *p* < 0.10; ** *p* < 0.01; * *p* < 0.05.

Treatment	Estimated Log Odds	Standard Error of Log Odds	Z-Value	Odds Ratio (OR)	OR Lower Confidence Interval	OR Upper Confidence Interval
Bb	0.24	0.12	1.97 *	1.27	1.00	1.60
Bm	0.00	0.07	0.03	1.00	0.87	1.16
F	0.18	0.10	1.79 +	1.20	0.98	1.46
T	0.23	0.08	2.74 **	1.25	1.07	1.48
Bb-	−0.14	0.20	−0.68	0.87	0.59	1.29
Bm-	−0.01	0.12	−0.04	0.99	0.78	1.27
F-	0.07	0.14	0.50	1.07	0.82	1.40
T-	−0.01	0.16	−0.06	0.99	0.72	1.37
Bb+	0.00	0.22	0.00	1.00	0.64	1.55
Bm+	−0.05	0.28	−0.18	0.95	0.55	1.65
F+	0.02	0.20	0.08	1.02	0.68	1.51
T+	−0.11	0.26	−0.45	0.89	0.54	1.48

## Data Availability

The raw data supporting the conclusions of this article will be made available by the authors on request.

## Supplementary Materials

The following supporting information can be downloaded at https://www.mdpi.com/article/10.3390/plants15131979/s1. Table S1: Means and standard deviations of morphological parameters (plant height (cm), number of leaves (pc), number of flowers (pc) and number of branches (pc)) of cucumber plants (n = 10) under *M. incognita* infection depending on biocontrol agents *Beauveria bassiana* (Bb), *Bacillus mojavensis* (Bm), *Fusarium proliferatum* (F), *Trichoderma asperellum* (T), and untreated control (C) treatment during the spring and summer experiments in 2023. Different letters show significant differences resulting from pairwise comparisons that were performed using Tukey’s HSD test or the Games–Howell test, conditioned on the homoscedasticity of each weekly subset as determined by Levene’s test (*p* < 0.05).
