# Supplementary material for: Evaluation of Biocontrol Agents Against Root-Knot Nematode (Meloidogyne incognita) in Cucumber (Cucumis sativus) Under Greenhouse Conditions
Source: Plants (Basel). 2026 Jun 26;15(13):1979. doi: 10.3390/plants15131979 (PMC13363713; doi:10.3390/plants15131979)
Supplement: Supplementary file 1 [file plants-15-01979-s001.zip › plants-4373466-supplementary_revised.pdf]

Table S1: Means and standard deviations of morphological parameters (plant height (cm), number of leaves (pc), number of flowers (pc) and number of branches (pc)) of cucumber plants (n = 10) under *M. incognita* infection depending on biocontrol agents *Beauveria bassiana* (Bb), *Bacillus mojavensis* (Bm), *Fusarium proliferatum* (F), *Trichoderma asperellum* (T), and untreated control (C) treatment during the spring and summer experiments in 2023. Different letters show significant differences resulting from pairwise comparisons that were performed using Tukey's HSD test or the Games–Howell test, conditioned on the homoscedasticity of each weekly subset as determined by Levene's test (p < 0.05).

Plant Height SPRING

	Spring Week1	Spring Week2	Spring Week3	Spring Week4	Spring Week5	Spring Week6	Spring Week7	Spring Week8	Spring Week9
Treatment	18 May 2023	24 May 2023	31 May 2023	06 June 2023	14 June 2023	21 June 2023	28 June 2023	10 July 2023	20 July 2023
Untreated control (C)	8.50 ± 0.96 a	10.52 ± 0.65 a	14.11 ± 2.03 a	20.83 ± 2.60 a	23.83 ± 4.66 a	27.10 ± 7.82 a	39.60 ± 11.64 a	76.60 ± 20.00 a	93.60 ± 24.77 a
Beauveria bassiana (Bb)	8.07 ± 1.57 a	10.15 ± 2.94 a	15.51 ± 5.00 a	25.10 ± 8.21 ab	26.30 ± 8.00 a	30.08 ± 12.35 a	47.10 ± 20.44 a	89.60 ± 27.42 a	109.60 ± 18.70 ab
Bacillus mojavensis (Bm)	9.28 ± 1.19 a	11.56 ± 1.04 a	17.63 ± 2.55 a	27.09 ± 4.44 b	29.20 ± 5.47 a	33.67 ± 11.76 a	55.55 ± 17.92 a	107.40 ± 27.18 a	134.00 ± 23.10 b
Fusarium proliferatum (F)	8.30 ± 1.33 a	10.11 ± 2.08 a	15.18 ± 3.03 a	24.33 ± 3.80 ab	26.50 ± 5.07 a	31.20 ± 10.42 a	49.30 ± 19.13 a	96.40 ± 27.51 a	128.80 ± 27.21 b
Trichoderma asperellum (T)	9.32 ± 1.91 a	11.21 ± 1.93 a	16.10 ± 2.88 a	23.53 ± 2.82 ab	25.10 ± 2.73 a	29.40 ± 10.06 a	48.40 ± 21.00 a	95.40 ± 34.24 a	120.42 ± 29.16 ab

Number of Leaves SPRING

Treatment	18 May 2023	24 May 2023	31 May 2023	06 June 2023	14 June 2023	21 June 2023	28 June 2023	10 July 2023	20 July 2023
Untreated control (C)	1.00 ± 0.00 a	2.00 ± 0.00 a	3.30 ± 0.48 a	3.80 ± 0.63 a	4.90 ± 0.74 a	7.00 ± 2.58 a	9.50 ± 2.37 a	12.20 ± 3.29 a	17.30 ± 3.56 a
Beauveria bassiana (Bb)	1.00 ± 0.00 a	2.70 ± 1.25 a	3.50 ± 0.53 a	4.70 ± 0.95 a	5.50 ± 1.18 a	7.90 ± 2.60 a	12.00 ± 3.62 a	15.50 ± 3.66 a	22.00 ± 3.59 ab
Bacillus mojavensis (Bm)	1.10 ± 0.32 a	2.10 ± 0.32 a	3.60 ± 0.52 a	4.60 ± 0.52 a	5.50 ± 0.85 a	8.20 ± 2.30 a	12.60 ± 2.91 a	15.80 ± 3.01 a	23.50 ± 3.21 b
Fusarium proliferatum (F)	1.00 ± 0.00 a	2.10 ± 0.32 a	3.50 ± 0.71 a	4.50 ± 0.71 a	5.30 ± 1.16 a	7.40 ± 2.27 a	11.70 ± 3.06 a	15.00 ± 3.92 a	23.40 ± 4.09 b
Trichoderma asperellum (T)	1.10 ± 0.32 a	2.10 ± 0.32 a	3.40 ± 0.52 a	4.60 ± 0.70 a	5.30 ± 1.34 a	7.90 ± 3.07 a	11.80 ± 4.02 a	15.20 ± 4.34 a	22.00 ± 5.37 ab

Number of Flowers SPRING

Treatment	21 June 2023	28 June 2023	10 July 2023	20 July 2023
Untreated control (C)	1.40 ± 1.07 a	4.50 ± 2.37 a	9.00 ± 3.33 a	9.00 ± 3.33 a
Beauveria bassiana (Bb)	1.80 ± 1.62 a	7.70 ± 3.86 a	11.30 ± 4.64 a	11.30 ± 4.64 a
Bacillus mojavensis (Bm)	2.10 ± 2.13 a	8.40 ± 4.17 a	14.70 ± 4.99 a	14.70 ± 4.99 a
Fusarium proliferatum (F)	1.60 ± 1.26 a	5.30 ± 3.23 a	12.60 ± 5.56 a	12.60 ± 5.56 a
Trichoderma asperellum (T)	0.80 ± 1.03 a	6.60 ± 3.89 a	11.20 ± 7.39 a	11.20 ± 7.39 a

Number of Branches SPRING

Treatment	21 June 2023	28 June 2023	10 July 2023	20 July 2023
Untreated control (C)	0.60 ± 0.84 a	2.00 ± 1.33 a	0.60 ± 1.26 a	0.60 ± 1.26 a
Beauveria bassiana (Bb)	0.70 ± 1.06 a	3.50 ± 2.55 a	1.20 ± 1.48 a	1.20 ± 1.48 a
Bacillus mojavensis (Bm)	1.10 ± 1.20 a	3.40 ± 2.01 a	2.10 ± 1.97 a	2.10 ± 1.97 a
Fusarium proliferatum (F)	1.20 ± 1.14 a	2.70 ± 1.64 a	2.30 ± 1.64 a	2.30 ± 1.64 a
Trichoderma asperellum (T)	1.20 ± 1.40 a	3.10 ± 1.85 a	1.50 ± 1.65 a	1.50 ± 1.65 a

Plant Height SUMMER

	Summer Week1	Summer Week2	Summer Week3	Summer Week4	Summer Week5	Summer Week6	Summer Week7	Summer Week8	Summer Week9
Treatment	31 July 2023	07 August 2023	16 August 2023	25 August 2023	30 August 2023	07 September 2023	15 September 2023	22 September 2023	29 September 2023
Untreated control (C)	7.21 ± 1.23 a	5.65 ± 0.57 a	9.26 ± 1.46 a	11.41 ± 1.89 a	16.43 ± 3.66 a	23.20 ± 7.86 a	33.00 ± 11.60 ab	36.40 ± 14.43 a	48.20 ± 17.73 a
Beauveria bassiana (Bb)	8.08 ± 1.88 a	6.21 ± 1.02 a	10.50 ± 2.13 a	13.34 ± 2.02 ab	18.57 ± 2.87 ab	25.70 ± 5.07 ab	37.20 ± 5.25 ab	50.70 ± 14.02 ab	69.00 ± 20.47 ab
Bacillus mojavensis (Bm)	8.90 ± 1.54 a	6.27 ± 0.82 a	11.25 ± 2.05 ab	15.22 ± 2.71 bc	20.99 ± 4.72 ab	31.35 ± 6.30 b	43.30 ± 9.80 b	54.50 ± 14.66 b	70.70 ± 21.85 ab
Fusarium proliferatum (F)	8.57 ± 1.88 a	5.92 ± 1.18 a	13.26 ± 2.65 b	17.01 ± 3.98 c	22.57 ± 4.80 b	31.10 ± 7.31 ab	35.90 ± 6.51 ab	57.50 ± 13.10 b	77.80 ± 21.03 b
Trichoderma asperellum (T)	7.93 ± 1.13 a	5.75 ± 1.52 a	10.95 ± 1.46 ab	13.51 ± 1.40 ab	17.96 ± 2.24 ab	27.20 ± 4.00 ab	31.40 ± 10.27 a	50.50 ± 11.21 ab	72.50 ± 17.42 ab

Number of Leaves SUMMER

Treatment	31 July 2023	07 August 2023	16 August 2023	25 August 2023	30 August 2023	07 September 2023	15 September 2023	22 September 2023	29 September 2023
Untreated control (C)	1.00 ± 0.00 a	2.20 ± 0.63 a	3.30 ± 0.48 a	4.70 ± 0.67 a	6.50 ± 1.18 a	8.30 ± 2.71 a	8.80 ± 2.70 a	11.60 ± 3.47 a	13.10 ± 3.38 a
Beauveria bassiana (Bb)	1.20 ± 0.42 a	2.70 ± 0.82 a	4.00 ± 0.67 ab	5.10 ± 0.74 ab	6.50 ± 1.18 a	10.00 ± 1.25 ab	11.30 ± 1.25 b	14.30 ± 1.16 ab	15.90 ± 1.37 ab
Bacillus mojavensis (Bm)	1.20 ± 0.42 a	2.70 ± 0.48 a	3.80 ± 0.42 ab	5.10 ± 0.57 ab	6.60 ± 0.84 a	10.20 ± 1.14 ab	12.00 ± 2.00 b	14.70 ± 2.21 b	16.50 ± 2.07 b
Fusarium proliferatum (F)	1.20 ± 0.42 a	2.80 ± 0.92 a	4.30 ± 0.67 b	5.60 ± 0.70 b	6.80 ± 1.75 a	10.50 ± 1.51 b	12.60 ± 1.07 b	15.50 ± 1.72 b	17.60 ± 2.12 b
Trichoderma asperellum (T)	1.10 ± 0.57 a	2.60 ± 0.97 a	3.90 ± 0.57 ab	5.10 ± 0.57 ab	7.10 ± 1.66 a	10.00 ± 0.47 ab	11.60 ± 1.07 b	15.00 ± 1.41 b	16.60 ± 1.65 b

Number of Flowers SUMMER

Treatment	16 August 2023	25 August 2023	30 August 2023	07 September 2023	15 September 2023	22 September 2023	29 September 2023
Untreated control (C)	1.20 ± 1.03 a	1.20 ± 1.03 a	4.90 ± 2.92 a	4.20 ± 1.69 a	5.30 ± 2.41 a	5.80 ± 2.70 a	5.40 ± 3.17 a
Beauveria bassiana (Bb)	2.20 ± 1.32 ab	2.20 ± 1.32 ab	6.10 ± 2.28 a	5.50 ± 2.12 a	8.60 ± 2.50 a	8.20 ± 2.94 ab	10.40 ± 6.13 a
Bacillus mojavensis (Bm)	3.40 ± 1.26 b	3.40 ± 1.26 b	7.80 ± 2.10 a	6.20 ± 2.15 a	8.30 ± 2.50 a	7.90 ± 2.69 ab	9.90 ± 3.41 a
Fusarium proliferatum (F)	3.40 ± 1.84 b	3.40 ± 1.84 b	8.10 ± 2.38 a	6.50 ± 2.27 a	8.80 ± 3.08 a	9.90 ± 3.60 b	9.70 ± 2.95 a
Trichoderma asperellum (T)	1.90 ± 1.10 ab	1.90 ± 1.10 ab	7.20 ± 2.90 a	5.80 ± 2.20 a	15.40 ± 22.12 a	9.20 ± 3.39 ab	10.30 ± 3.33 a

Number of Branches SUMMER

Treatment	29 September 2023
Untreated control (C)	0.10 ± 0.32 a
Beauveria bassiana (Bb)	0.20 ± 0.63 a
Bacillus mojavensis (Bm)	0.20 ± 0.63 a
Fusarium proliferatum (F)	0.60 ± 1.07 a
Trichoderma asperellum (T)	0.30 ± 0.48 a
